# Molecular Characterization of *Trypanosoma cruzi* SAP Proteins with Host-Cell Lysosome Exocytosis-Inducing Activity Required for Parasite Invasion

**DOI:** 10.1371/journal.pone.0083864

**Published:** 2013-12-31

**Authors:** Tamiris Zanforlin, Ethel Bayer-Santos, Cristian Cortez, Igor C. Almeida, Nobuko Yoshida, José Franco da Silveira

**Affiliations:** 1 Departamento de Microbiologia, Imunologia e Parasitologia, Escola Paulista de Medicina, Universidade Federal de São Paulo, São Paulo, São Paulo, Brazil; 2 Department of Biological Sciences, The Border Biomedical Research Center, University of Texas at El Paso, El Paso, Texas, United States of America; State University of Campinas, Brazil

## Abstract

**Background:**

To invade target cells, *Trypanosoma cruzi* metacyclic forms engage distinct sets of surface and secreted molecules that interact with host components. Serine-, alanine-, and proline-rich proteins (SAP) comprise a multigene family constituted of molecules with a high serine, alanine and proline residue content. SAP proteins have a central domain (SAP-CD) responsible for interaction with and invasion of mammalian cells by metacyclic forms.

**Methods and Findings:**

Using a 513 bp sequence from SAP-CD in blastn analysis, we identified 39 full-length SAP genes in the genome of *T. cruzi*. Although most of these genes were mapped in the *T. cruzi in silico* chromosome TcChr41, several SAP sequences were spread out across the genome. The level of SAP transcripts was twice as high in metacyclic forms as in epimastigotes. Monoclonal (MAb-SAP) and polyclonal (anti-SAP) antibodies produced against the recombinant protein SAP-CD were used to investigate the expression and localization of SAP proteins. MAb-SAP reacted with a 55 kDa SAP protein released by epimastigotes and metacyclic forms and with distinct sets of SAP variants expressed in amastigotes and tissue culture-derived trypomastigotes (TCTs). Anti-SAP antibodies reacted with components located in the anterior region of epimastigotes and between the nucleus and the kinetoplast in metacyclic trypomastigotes. In contrast, anti-SAP recognized surface components of amastigotes and TCTs, suggesting that SAP proteins are directed to different cellular compartments. Ten SAP peptides were identified by mass spectrometry in vesicle and soluble-protein fractions obtained from parasite conditioned medium. Using overlapping sequences from SAP-CD, we identified a 54-aa peptide (SAP-CE) that was able to induce host-cell lysosome exocytosis and inhibit parasite internalization by 52%.

**Conclusions:**

This study provides novel information about the genomic organization, expression and cellular localization of SAP proteins and proposes a triggering role for extracellular SAP proteins in host-cell lysosome exocytosis during metacyclic internalization.

## Introduction

*Trypanosoma cruzi* is the etiological agent of Chagas disease (also known as American trypanosomiasis), a potentially life-threatening illness that affects approximately 10 million people in the world and accounted for 10,000 deaths in 2008 [1]. The vast majority of *T. cruzi-*infected individuals live in Latin America, but the disease has also spread to non-endemic regions in the United States, Europe, Australia, Canada and Japan because of population migration and may pose a new worldwide health problem [2]. *T. cruzi* has a complex life cycle involving vertebrate and invertebrate hosts. Epimastigote forms replicate in the triatomine insect vectors. On reaching the final portion of the triatomine digestive tract, they transform into metacyclic trypomastigotes, which are released in the feces during bloodmeals and can be transmitted through the bite wound or ocular mucosa. Infection by metacyclic forms, which can also occur through the oral route, starts when the parasite adheres to and invades host cells. Metacyclic forms differentiate into amastigotes, which transform into trypomastigotes upon intracellular replication. These parasite forms, which are released into the circulation when the host cell ruptures and disseminate to diverse organs and tissues, can be transmitted by blood transfusion.

Most nucleated mammalian cells are susceptible to *T. cruzi* invasion, a process that is distinct from classical phagocytosis and involves Ca^+2^-dependent F-actin disruption and lysosome exocytosis, both of which contribute to the formation of parasitophorous vacuoles [3], [4]. The metacyclic stage-specific surface glycoprotein GP82, which is implicated in host-cell invasion of highly infective *T. cruzi* strains, is a cell adhesion molecule that binds to host cells [5], [6] and induces Ca^+2^ mobilization and lysosome exocytosis [7], [8]. Molecules secreted by *T. cruzi* into the extracellular medium may also participate in the process of parasite internalization. Cruzipain, a cysteine protease expressed in all parasite developmental forms [9], [10], is constitutively secreted by trypomastigotes. According to Scharfstein et al. [11], cruzipain cleaves the cell-bound kininogen, generating bradykinin, which binds to the bradykinin receptor (B_2_R) and triggers IP_3_-mediated Ca^+2^ influx. Vesicles released by *T. cruzi*, either as exosomes or plasma membrane-derived vesicles that can contain lipids, proteins and nucleic acids [12], [13], have been implicated in infection by this parasite. Virulence factors, such as GP82 and cruzipain, were detected in *T. cruzi* vesicles [14], and inoculation of trypomastigote membrane vesicles into mice was found to stimulate the production of cytokines such as IL-4 and IL-10 that modulate infection [15].

SAP (serine-, alanine- and proline-rich) proteins, which are released by *T. cruzi* metacyclic forms into the extracellular medium, have been implicated in mammalian cell invasion [16]. Members of the SAP multigene family are characterized by a high serine (7.2 to 11.7%), alanine (12.2 to 17.3%) and proline (7.06 to 13.5%) residue content [17]. SAP genes have been classified into four groups (SAP1 to SAP4) according to the presence of an endoplasmic reticulum (ER) and/or glycosylphosphatidylinositol (GPI) anchor-addition signal peptide(s). Most of the SAP genes encode both an N-terminal signal peptide and a C-terminal GPI anchor addition site (SAP1 group). SAP binds to the host cell through its central domain (SAP-CD) and triggers intracellular Ca^+2^ mobilization [16]. In the present study we present further characterization of SAP proteins, including their genomic distribution, expression and cellular localization. We also shed light on the mechanism of action of SAP in host-cell invasion by metacyclic trypomastigotes.

## Materials and Methods

### Ethics Statement

All experiments involving animal work were conducted under Brazilian National Committee on Ethics Research (CONEP) ethic guidelines, which are in accordance with international standards (CIOMS/OMS, 1985). The protocol was approved by the Committee on Ethics of Animal Experiments of Universidade Federal de São Paulo (Permit Number: CEP 0913/10). During the experimental procedures, all efforts were made to minimize animal suffering.

### Parasites and mammalian cell culture

*T. cruzi* epimastigotes (clones CL Brener, Dm28c and the CL strain) were maintained cyclically in mice and in liver infusion tryptose (LIT) medium containing 10% fetal bovine serum (FBS). To obtain metacyclic forms, epimastigotes were grown for one passage in Grace's medium (Gibco). Metacyclic trypomastigotes were obtained from cultures in the stationary growth phase and were purified by passage through DEAE-cellulose column, as described previously [18]. HeLa and Vero cells were grown at 37°C in Dulbecco's modified Eagle medium (DMEM, Gibco) supplemented with 10% FBS in humidified 5% CO_2_ atmosphere. Vero cells infected with metacyclic trypomastigotes (CL strain) were maintained in DMEM supplemented with 2.5% FBS until release of tissue culture-derived trypomastigotes (TCTs). To obtain extracellular amastigotes, TCTs isolated from the supernatant of infected Vero cell cultures were incubated in LIT medium pH 5.8 for 24 h at 37°C.

### Sequence similarity search

A 513 bp sequence from the central domain of SAP (SAP-CD, accession number AF199419) was used to identify SAP genes in the genome of *T. cruzi* clone CL Brener (available in TriTrypDB database, http://tritrypdb.org/tritrypdb) by the blastn algorithm [19]. Sequences with alignment >250 bp and similarity >65% were included in the analysis.

### Separation of *T. cruzi* chromosomal DNA by pulsed-field gel electrophoresis (PFGE) and chromoblot hybridization

Chromosomal DNA from clone CL Brener and CL strain were isolated as described [20] and separated by PFGE in 1.2% agarose gels diluted in 0.5× TBE (45 mM Tris/45 mM boric acid/1 mM EDTA pH 8.3) using Gene Navigator System (Amersham Pharmacia Biotech, USA) and following the conditions described by Cano et al. [21]. Gels were stained with ethidium bromide (0.5 µg/mL) and transferred to nylon filters using Vacuum Gene XL System (Pharmacia). Nylon membranes were pre-hybridized at 42°C for 1 h (50% formamide/5× SSC/5× Denhart's solution/0.1 mg/mL salmon sperm DNA) and hybridized overnight at 42°C with the ^32^P-labeled SAP-CD fragment. Following hybridization, membranes were washed twice (30 min each wash at 42°C) in 2× SSC containing 0.1% SDS and 0.1% sodium pyrophosphate and then underwent one additional wash (30 min at 56°C) in 0.1× SSC containing 0.1% SDS and 0.1% sodium pyrophosphate. Membranes were then exposed to X-ray films.

### Cloning of SAP genes by reverse transcriptase PCR

Total RNA from epimastigotes (5.0×10^7^ cells), metacyclic trypomastigotes (1.0×10^8^ cells) and extracellular amastigotes (5.0×10^7^ cells) derived from the CL strain was extracted using TRIzol®, and first-strand cDNA was synthesized using ThermoScript™ RT-PCR System according to the manufacturer's instructions (Invitrogen). Before cDNA synthesis, total RNA was treated with DNaseI (Invitrogen). The efficiency of the DNAseI treatment method to remove genomic DNA from RNA samples was assessed by conventional PCR using specific primers that amplified the tubulin gene. No amplification was detected in the RNA samples after treatment with DNAseI, indicating the absence of DNA contaminants. Transcription of SAP genes was analyzed by RT-PCR using epimastigote, metacyclic trypomastigote or amastigote cDNA as a template and 5′-GCTCCCCTTTCCTCTGCG-3′ and 5′-TCAGCCCAGTGTCCCGTA-3′ primers to amplify a conserved 135 bp fragment shared by all full-length copies of SAP genes. Alternatively, 5′-ATGCGCCGTGTGTTTTGTGTC-3′ and 5′-TCAGCCCAGTGTCCCGTA-3′ primers were used to amplify the entire open reading frames of SAP genes. Amplified PCR products were cloned into pGEM®-T easy vector (Promega) and the nucleotide sequences of cDNA recombinant clones were determined using the dideoxynucleotide chain termination method with BigDye Terminator cycle sequencing chemistry in an ABI PRISM 3100 sequencer (Applied Biosystems).

### Real Time PCR (qRT-PCR)

The same primers used to amplify a conserved 135 bp fragment shared by all SAP genes were used in qRT-PCR. Primers that amplify the GAPDH constitutive gene (5′- TGGAGCTGCGGTTGTCATT-3′ and 5′-AGCGCGCGTCTAAGACTTACA-3′) were used as an endogenous control. Reactions were performed in triplicate with 500 nM forward and reverse SAP primers or 200 nM forward and reverse GAPDH primers, SYBR Green Master Mix (Applied Biosystems) and epimastigote or metacyclic trypomastigote cDNA synthesized as described above. Reactions were carried out in an ABI PRISM 7000 (Applied Biosystems) thermocycler following standard cycling conditions. The data were analyzed by the -2^ΔΔCT^ method after normalization with GAPDH using 7000 SDS software (Applied Biosystems).

### Construction of the plasmid pTREX/SAP_GFP and parasite transfection

To construct the plasmid pTREX/SAP_GFP, the entire open reading frame of a SAP gene (accession number Tc00.1047053507163.30) was cloned in frame with the green fluorescent protein (GFP) in the pTREX vector [22]. Two PCR amplifications were carried out in parallel using primers that contained artificial restriction enzyme sites (represented in bold) (SAP: 5′-**GAATTC**ATGATGCGCCGTGTGTTTTGTGTCGTGTTGGC-3′ and 5′-**CGGCCGC**TCAGCCCAGTGTCTCGTACGCAAGGACAGCCAGAACAAGCAGCA-3′; GFP: 5′**-GCATGC**TGGTGAGCAAGGGCGAG-3′ and 5′**-GCATGC**CGTACAGCTCGTCCATGCCGAG-3′). As a template, we used genomic DNA of clone CL Brener or pTREX/GFP vector [22]. Initially, the PCR products were individually cloned into pGEM®-T easy vector (Promega). The plasmid pGEM-T easy/GFP was digested with Sph1, and the GFP_Sph1 fragment was cloned into the plasmid pGEM-T easy/SAP previously digested with the same enzyme. The plasmid pGEM-T easy/SAP_GFP was then digested with EcoRI and NotI, and the SAP_GFP fragment was cloned into the pTREX vector previously digested with the same enzymes. Epimastigotes from the CL strain (1.0×10^8^) were washed in electroporation buffer (NaCl 137 mM/HEPES 21 mM/Na_2_HPO_4_ 5.5 mM/KCl 5 mM/glucose 0.77 mM pH 7.0) by centrifugation at 6 000 *g* for 5 min and resuspended in 360 µL of electroporation buffer and 40 µL of the plasmid pTREX/SAP_GFP or the control pTREX/GFP (100 µg). Transfection was performed using two pulses of 450 V and 500 µF in a Gene Pulser Xcell Total System (Bio-Rad). After electroporation, cells were recovered in 5 mL LIT supplemented with 20% FBS at 28°C. For selection of the transfected parasites, 500 µg/mL of G418 (Sigma) was added 48 h after transfection.

### Immunofluorescence microscopy of parasites

Epimastigotes, metacyclic trypomastigotes, extracellular amastigotes and tissue culture-derived trypomastigotes (CL strain) were washed in PBS, fixed for 30 min in 4% paraformaldehyde diluted in PBS and air dried on glass slides. Slides were then washed once in PBS, permeabilized with PBS containing 1% saponin for 30 min, blocked for 1 h in PBS containing 10% BSA and incubated with anti-SAP antibodies (diluted 1∶50 in PBS) and with Alexa Fluor 488-conjugated anti-rabbit IgG (diluted 1∶200) containing 10 µg/mL DAPI (4′,6′-1-diamino-2-phenylindole dihydrochloride). For colocalization assays, live epimastigotes were incubated for 30 min in a buffer containing 116 mM NaCl, 5.4 mM KCl, 0.8 mM MgSO_4_, 50 mM HEPES (pH 7.4) and 5 µg of concanavalin_A-TRITC/mL at room temperature, as previously described by Cuevas et al. [23]. Cells were then fixed and processed as described above. Alternatively, extracellular amastigotes were incubated with MAb-2C2 (diluted 1∶100 in PBS) for 1 h, following 1-h incubation with Alexa Fluor-568-conjugated anti-mouse IgG (diluted 1∶200). Slides were observed under a fluorescence microscope (Olympus BX51) and images were acquired with an Olympus DP71 CCD camera using Image Pro Plus 6.2 software (Media Cybernetic). Epimastigotes and extracellular amastigotes transfected with the plasmid pTREX/SAP_GFP or the control pTREX/GFP were labeled with an anti-GFP monoclonal antibody (Sigma, diluted 1∶100) following the same protocol described above.

### Preparation of parasite conditioned medium (CM)

Metacyclic trypomastigotes (CL strain) were incubated in PBS (1.0×10^8^ cells/mL) for 16 h at 28°C. Parasites were centrifuged at 3 000 *g* for 10 min and parasite-free supernatants were filtered with 0.22-µm syringe filter (Millipore). Vesicle and soluble-protein fractions were obtained from epimastigotes and metacyclic trypomastigotes (Dm28c) conditioned medium as previously described [14]. Briefly, epimastigotes were harvested from exponentially growing cultures, washed in DMEM without FBS and incubated in the same medium at a concentration of 1.0×10^8^ cells/mL for 6 h at 28°C. Metacyclic trypomastigote forms were incubated in TAU3AAG medium at a concentration of 1.0×10^8^ cells/mL for 6 h at 28°C. Parasite viability was assessed by propidium iodide incorporation and more than 98% of cells were viable at the end of the incubation period. Following 6-h incubation, parasites were removed by centrifugation at 3 000 *g* for 10 min at 4°C. The cell-free supernatant was filtered in 0.45-µm syringe filter (Millipore), transferred to 13.2-mL polyallomer tube, and centrifuged at 100 000 *g* for 2 h at 4°C to obtain the first pellet, enriched in plasma membrane-derived vesicles (V2). The resulting supernatant was transferred to another polyallomer tube and then centrifuged at 100 000 *g* for 16 h at 4°C, to obtain the second pellet, enriched in exosomes (V16) and soluble proteins in the vesicle-free supernatant (VF).

### Mass spectrometry analysis

Raw data derived from Bayer-Santos et al. [14] was obtained from proteomecommons.org and analyzed as previously described [14] with minor modifications. Briefly, all spectra obtained were searched using Sequest (version v.27; Thermo Fisher Scientific) and X! Tandem (version 2007.01.01.2; http://www.thegpm.org/tandem/) algorithms against sequences from *Trypanosoma* spp., BSA, human keratin and porcine trypsin (downloaded from GenBank on October 10, 2011). Parameters for the database search were as follows: trypsin cleavage at both termini and two missed cleavages allowed; 1 Da for peptide mass tolerance; 1 Da for fragment mass tolerance; and cysteine carbamidomethylation and methionine oxidation as fixed and variable modifications, respectively. The Scaffold platform (version 3.4.3; Proteome Software, Portland, OR) was used to validate peptide and protein identifications. As Bayer-Santos et al. [14] only validated proteins identified by two distinct peptides, we searched for SAP proteins among those data identified by only one peptide. All SAP spectra found were manually validated and accepted only if the protein identification probability was greater than 90% for peptides and 90% for proteins; Xcorr (CrossCorr/avg [AutoCorr offset = −75 to 75])≥1.5, 2.0 and 2.5, for singly, doubly and triply charged peptides, respectively; and DC*n* (Xcorr_1_ – Xcorr_2_/Xcorr_1_)≥0.1.

### SDS-PAGE and western blot assays

Protein extracts from epimastigotes (3.0×10^7^ cells), metacyclic trypomastigotes (1.0×10^8^ cells), extracellular amastigotes (3.0×10^7^ cells) and tissue culture-derived trypomastigotes (1.0×10^8^ cells) derived from CL strain and parasite conditioned medium prepared as described above were separated by SDS-PAGE in a 12% polyacrylamide gel and transferred to nitrocellulose membranes. Western blot assays were carried out with MAb-SAP diluted 1∶100 in PBS containing 5% fat-free milk (PBS/milk 5%) and anti-mouse IgG peroxidase conjugate (Invitrogen) diluted 1∶5000 in PBS/milk 5%. The reaction was revealed by chemiluminescence using the ECL Western blot detection reagents and Hyperfilm MP (GE Healthcare). Alternatively, protein extracts from epimastigotes transfected with the plasmid pTREX/SAP_GFP or the control pTREX/GFP were reacted with anti-GFP monoclonal antibody (Sigma, diluted 1∶500) or MAb-SAP (diluted 1∶100) following the same protocol described above.

### Expression and purification of central domain SAP-CD recombinant proteins

The constructs SAP-NT, SAP-CE and SAP-CT, which contain the amino-terminal, central and carboxy-terminal regions of the SAP central domain (SAP-CD) in frame with glutathione S-transferase (GST), were generated by PCR using primers that contained artificial restriction enzyme sites (represented in bold) (SAP-NT: 5′-CCG**GAATTC**ATCATGATTTGCGACATG-3′ and 5′-CCG**GAATTC**ATCAACAGAGTTAGCACC-3′; SAP-CE: 5′-CCG**GAATTC**ATCATGAAACTCCGGAA-3′ and 5′-CCG**GAATTC**ATCAACAGAGTTAGCACC-3′; SAP-CT: 5′-CCG**GAATTC**ATCATGAAACTCCGGAA-3′ and 5′-CCG**GAATTC**ATCTAGAGATTCGGATGC-3′). As a template, we used the plasmid pGEX1λT/SAP-CD. The amplified PCR products were cloned into pGEX1λT/EcoRI vector (GE Healthcare).

The plasmid constructions were transformed into DH5-α bacterial cells, which were grown in LB (Luria Bertani) medium for 16 h at 37°C. Expression of recombinant proteins was induced with 1 mM isopropyl-β-D-thiogalactopyranoside for 4 h at 37°C, followed by centrifugation at 12 000 *g* for 10 min at 4°C. After resuspension in PBS with a protease inhibitors cocktail (Santa Cruz), the pellet was sonicated and centrifuged at 12 000 *g* for 15 min at 4°C and the resultant supernatant was passed through prepacked Glutathione Sepharose 4B columns (GE Healthcare). To check that the desired protein had been obtained, the purified samples were analyzed by SDS-PAGE stained with Coomassie blue. Purified proteins were quantified by reaction with Coomassie Plus Assay Reagent in 96-well plates and reading at 620 nm. The purified recombinant proteins SAP-NT, SAP-CE and SAP-CT were tested in western blot assays using MAb-SAP (diluted 1∶100) and anti-mouse IgG peroxidase conjugate (diluted 1∶5000).

### Host-cell invasion assay

Cell invasion assays were carried out as previously described [24]. HeLa cells (1.5×10^5^) cultured for 16 h at 37°C in 24-well plates containing 13-mm diameter round glass coverslips were incubated with or without the purified recombinant protein SAP-CE (40 μg/mL) or GST (40 μg/mL) for 30 min before addition of metacyclic forms (3.0×10^6^). After incubation for 1 h, the duplicate coverslips were washed in PBS, fixed in Bouin solution, stained with Giemsa and sequentially dehydrated in acetone, a graded series of acetone:xylol (9∶1, 7∶3, 3∶7) and xylol. The number of intracellular parasites was counted in 500 cells.

### Exocytosis assay

Exocytosis assays were performed as previously described [7]. Semi-confluent HeLa cells grown in 24-well plates in DMEM without phenol red were incubated for 1 h with or without the purified recombinant protein SAP-CE (20 µg/mL). After supernatants were collected, cells were lysed in DMEM containing 1% NP-40, and 30 µL of 1 M sodium acetate pH 4.0 was added to lower the pH. Following centrifugation at 13 000 *g* for 5 min, supernatants were collected and 20-µL aliquots were diluted in 60 µL citrate buffer and 160 µL of 100 mM 4-nitrophenyl N-acetyl-β-D-glucosaminide (Sigma). After incubation for 1 h at 37°C, reaction was stopped by adding 720 µL of 200 mM sodium borate pH 9.8 and absorbance was measured at 405 nm in a Labsystems Multiskan MS plate reader. Exocytosis was expressed as a percentage of total β-hexosaminidase activity (supernatant + cell extract).

### Immunofluorescence microscopy for visualization of lysosomes

HeLa cells were incubated for 1 h with or without 20 µg/mL of the purified recombinant protein SAP-CE or GST. After fixation for 30 min with 4% paraformaldehyde diluted in PBS, cells were incubated with 50 mM NH_4_Cl in PBS for 30 min, washed three times in PBS and incubated for 1 h at room temperature with mouse anti-human Lamp-2 antibody (H4B4 monoclonal antibody) diluted 1∶4 in PGN (0.15% gelatin in PBS containing 0.1% sodium azide) and 1% saponin. Following three washes in PBS, cells were incubated for 1 h in PGN with Alexa Fluor 488-conjugated anti-mouse IgG (Invitrogen) diluted 1∶250, 100 ng/mL phalloidin-TRITC and 10 µg/mL DAPI. Coverslips were mounted on glass slides and examined under a fluorescence microscope (Olympus BX51). Images were acquired with an Olympus DP71 CCD camera using Image Pro Plus 6.2 software (Media Cybernetic).

### Statistics

GraphPad InStats program was used to determine significance by Student's *t-*test.

## Results and Discussion

### SAP genes are widespread in *T. cruzi* genome

Recently, contigs generated in the sequencing of the *T. cruzi* (clone CL Brener) genome were assembled into 41 chromosome-sized scaffolds (TcChr) by Weatherly et al. [25] (available in the TriTrypDB database). In a previous work, Baida et al. [16] had identified 39 full-length copies of SAP genes in the genome of clone CL Brener. Here, we decided to revisit these data and allocate SAP sequences in the 41 TcChr. To this end, we used a 513 bp sequence from the central domain of SAP genes (SAP-CD) as a query in blastn analysis against *T. cruzi* TriTrypDB database. Confirming previous results [16], of 51 SAP sequences identified 39 corresponded to full-length copies of SAP genes and 12 to truncated sequences. The size of full-length SAP genes varied from 813 to 1740 bp (Table S1). Approximately 50% of the SAP sequences (26/51) were allocated in TcChr41 (22 full-length genes and 4 truncated sequences), and 14 SAP sequences (12 full-length copies and 2 truncated sequences) were distributed in chromosomes TcChr18, 38, 40, 20 and 16 (Table S2). The remaining 11 SAP sequences (5 full-length genes and 6 truncated sequences) were found in non-allocated contigs. In summary, our results showed that SAP genes were distributed among at least six different *T. cruzi* TcChr.

**Figure 1:**
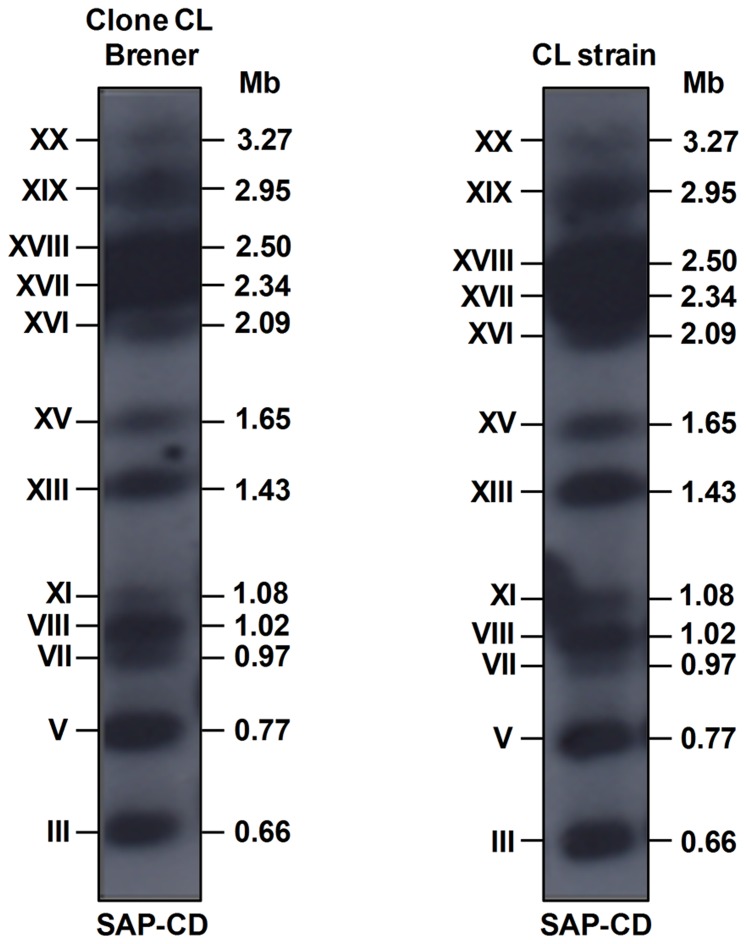
Mapping of SAP genes in the chromosomal bands of *T. cruzi*. Chromosomal bands of clone CL Brener and the CL strain were separated by PFGE, stained with ethidium bromide, transferred to nylon membranes and hybridized with the SAP-CD ^32^P-labeled 513 bp fragment. The sizes of the chromosomal bands are shown in Mb on the right, and the chromosomal-band nomenclature described by Cano et al. [21] is shown on the left in Roman numerals.

We hybridized chromosomal DNA bands of clone CL Brener and CL strain with the 513 bp SAP-CD fragment, the same sequence used as a query to search for SAP genes in the TriTrypDB database. The probe hybridized with twelve chromosomal bands (XX, XIX, XVIII, XVII, XVI, XV, XIII, IX, VIII, VII, V and III) in both isolates (Figure 1), confirming that SAP genes are spread out across the genome. Our chromoblot results are in agreement with Souza et al. [20], who mapped TcChr38 and TcChr40/TcChr16 in the clone CL Brener chromosomal bands XIX and XVI, respectively. Additionally, we found that TcChr41 maps in the clone CL Brener chromosomal band XVIII, which exhibited an intense hybridization signal in chromoblot analysis (Figure 1) and contains almost 50% of the SAP genes identified in the *T. cruzi* genome (Table S2). As expected, the number of chromosomal bands identified by chromoblot analysis was much higher than the number of TcChr containing SAP genes, since a considerable number of SAP sequences were distributed in non-allocated contigs. It is worth mentioning that the number of SAP sequences in *T. cruzi* genome may be even larger than that obtained by bioinformatics analysis, since the alignment of all the contigs derived from genome sequencing of clone CL Brener [26] is only partially complete and is represented by redundant sequences, underestimating the real number of genes and pseudogenes.

All the genes present in 100 kb genomic regions (TriTrypDB database) containing each of the 39 full-length SAP genes were annotated. Confirming previous studies by Baida et al. [16], who mapped the genes present in 10 kb genomic regions (GeneDB database), the analyzed genomic regions were found to be rich in multigene families that encode surface antigens such as mucin-associated proteins (MASP), mucins, *trans*-sialidases (TS) and surface proteases GP63 (Table S3), a feature characteristic of the *T. cruzi* genome. These multigene families, together with repetitive sequences (micro- and minisatellites, retrotransposons and subtelomeric repeats), correspond to 50% of the genome of clone CL Brener [26]. SAP genes constitute a moderately repeated (30–50 copies) multigene family that can be compared with the glycosyltransferases, RNA helicase (eIF-4a), protein kinases (NEK group) and MASP-related protein families [26].

### SAP genes are differentially expressed in the developmental forms of *T. cruzi*

The expression of SAP genes during *T. cruzi* life cycle was investigated by performing RT-PCR with primers that amplified a conserved 135 bp fragment shared by the 39 full-length SAP genes. Amplification of the 135 bp fragment was detected in epimastigotes, metacyclic trypomastigotes and extracellular amastigotes (data not shown), indicating that at least one SAP gene is transcribed in all developmental forms. To further investigate the repertoire of SAP transcripts, we designed primers that amplified the entire open reading frame including the sequences encoding the N-terminal signal peptide and those encoding the C-terminal GPI anchor addition site in SAP genes. The full-length transcripts were cloned and sequenced. Eight, six and five different SAP transcripts were isolated from epimastigotes, metacyclic trypomastigotes and extracellular amastigotes, respectively, which four of them were shared by the three developmental forms analyzed (Table 1).

**Table 1:** SAP transcripts isolated from epimastigotes, metacyclic trypomastigotes and extracellular amastigotes of the *T. cruzi* CL strain by RT-PCR amplification.

Developmental form of the parasite	Accession number^(1)^	TcChr^(2)^
Epimastigotes	Non-annotated^(3)^	Tcruzi_21632
Epimastigotes	Tc00.1047053510013.200^(3)^	41
Epimastigotes	Tc00.1047053508219.90^(3)^	41
Epimastigotes	Tc00.1047053510021.80^(3)^	41
Epimastigotes	Tc00.1047053506667.30	20
Epimastigotes	Tc00.1047053511487.150	41
Epimastigotes	Tc00.1047053508871.51	41
Epimastigotes	Tc00.1047053507953.70	41
Metacyclic trypomastigotes	Non-annotated^(3)^	Tcruzi_21632
Metacyclic trypomastigotes	Tc00.1047053510013.200^(3)^	41
Metacyclic trypomastigotes	Tc00.1047053508219.90^(3)^	41
Metacyclic trypomastigotes	Tc00.1047053510021.80^(3)^	41
Metacyclic trypomastigotes	Tc00.1047053506499.190	41
Metacyclic trypomastigotes	Tc00.1047053508221.260	41
Extracellular amastigotes	Non-annotated^(3)^	Tcruzi_21632
Extracellular amastigotes	Tc00.1047053510013.200^(3)^	41
Extracellular amastigotes	Tc00.1047053508219.90^(3)^	41
Extracellular amastigotes	Tc00.1047053510021.80^(3)^	41
Extracellular amastigotes	Tc00.1047053508247.1104^(4)^	18
	Tc00.1047053511233.160^(4)^	
	Tc00.1047053508853.30^(4)^	

1) Accession number according to the TriTrypDB database.

(2) Localization of SAP genes based on the 41 chromosome-sized scaffolds [25].

(3) SAP transcripts shared by epimastigotes, metacyclic trypomastigotes and extracellular amastigotes.

(4) The accession numbers represent the three copies of the same gene.

**Figure 2:**
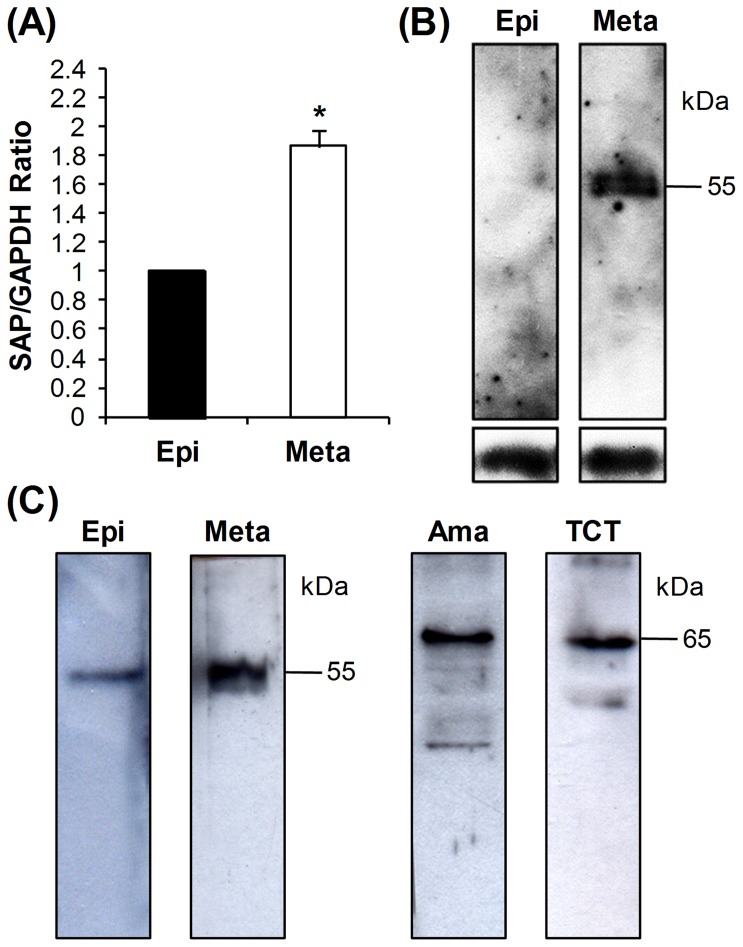
Expression of SAP proteins in the developmental forms of the *T. cruzi* CL strain. (A) The levels of SAP transcripts in epimastigotes (Epi) and metacyclic trypomastigotes (Meta) were estimated by qRT-PCR using primers that amplified a conserved 135 bp fragment shared by all SAP genes. The values, which were calculated after normalization with GAPDH transcripts and using epimastigotes as the reference sample (SAP/GAPDH ratio  = 1), are the means ± standard deviations of four independent experiments performed in triplicate. The difference between epimastigotes and metacyclic trypomastigotes was significant (*p<0.0001). (B) SAP expression was determined by quantitative western blot using total protein extracts from epimastigotes and metacyclic trypomastigotes (15 µg protein/lane) reacted with MAb-SAP (diluted 1∶100). As loading control, α-tubulin was used. (C) Difference in size of SAP variants expressed in the different *T. cruzi* developmental forms. Total protein extracts from epimastigotes (3.0××10^7^ cells), metacyclic trypomastigotes (1.0×10^8^ cells), extracellular amastigotes (3.0×10^7^ cells) and tissue culture-derived trypomastigotes (1.0×10^8^ cells) were separated by SDS-PAGE, transferred to nitrocellulose membranes and incubated with MAb-SAP (diluted 1∶100). The relative molecular masses (kDa) of the immunoreactive proteins are shown on the right.

As previous data suggested that SAP proteins play a role in mammalian cell adhesion and invasion by metacyclic trypomastigotes [16], the profile of SAP transcripts in these parasite forms was further analyzed and compared with that of noninvasive epimastigotes by real-time PCR. The level of SAP transcripts in metacyclic forms was about twice as high as that in epimastigotes used as reference (SAP/GAPDH ratio  = 1) (Figure 2A), supporting the idea that the expression levels of genes related to events such as host-cell adhesion/invasion are higher in infective forms. Expression of SAP proteins was analyzed by western blot using the anti-SAP monoclonal antibody (MAb-SAP) produced in mouse against the purified recombinant protein SAP-CD. In accordance with quantitative real-time PCR results, SAP expression was higher in metacyclic trypomastigotes when compared with epimastigotes (Figure 2B).

To analyze the size of the SAP variants expressed in each developmental stage we performed a qualitative western blot in which different amount of cells were used in order to obtain a detectable signal. In epimastigotes and metacyclic trypomastigotes, a major 55 kDa protein was detected (Figure 2C). In contrast, in extracellular amastigotes and tissue culture-derived trypomastigotes (TCTs), MAb-SAP reacted with a major 65 kDa SAP protein and with other bands of weaker intensity ranging from 40 to 60 kDa (Figure 2C). These results suggest that the expression of different SAP variants or post-translational modifications vary during *T. cruzi* life cycle according to the parasite developmental stages found in the insect or mammalian host.

### SAP proteins are differentially distributed in the developmental forms of *T. cruzi*

**Figure 3:**
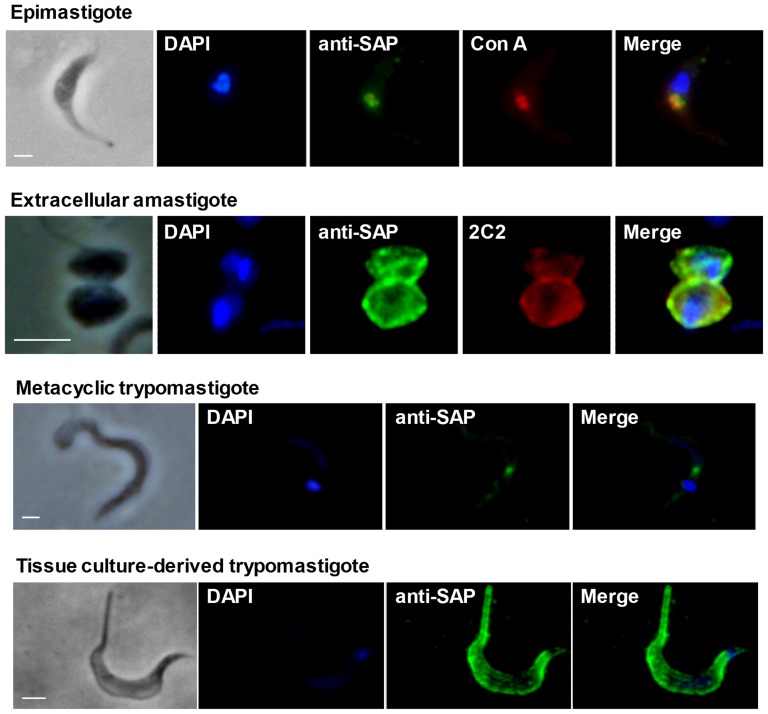
Cellular distribution of SAP proteins in the developmental forms of the *T. cruzi* CL strain. Epimastigotes, metacyclic trypomastigotes, extracellular amastigotes and tissue culture-derived trypomastigotes were fixed with 4% paraformaldehyde, permeabilized with saponin and incubated with anti-SAP polyclonal antibodies diluted 1∶50, followed by incubation with Alexa Fluor 488-conjugated anti-rabbit IgG (green). The figure also shows the colocalization of anti-SAP with concanavalin_A in epimastigotes (red) and with MAb-2C2 in extracellular amastigotes (red). Parasite DNA was stained with DAPI (blue). Scale bar, 5 µm.

The cellular localization of SAP proteins was determined by immunofluorescence microscopy using anti-SAP polyclonal antiserum (anti-SAP) produced in rabbit against the recombinant protein SAP-CD. Anti-SAP antibodies were used because of the weak reactivity of MAb-SAP in immunofluorescence assays. In epimastigotes, SAP proteins were concentrated in the anterior region of the parasite (Figure 3). We also demonstrated that anti-SAP colocalizes with concanavalin_A (Figure 3), which was previously shown to react with N-glycosylated proteins on the surface of epimastigotes as well as in the cytostome [27]. In metacyclic trypomastigotes, anti-SAP recognized components located between the nucleus and the kinetoplast (Figure 3). By contrast, extracellular amastigotes and tissue culture-derived trypomastigotes were labeled on the cell surface (Figure 3), suggesting that these parasite forms not only produce distinct sets of SAP proteins (Figure 2C), but direct them to different cellular locations. Confirming the localization of SAP proteins in the external face of the amastigote plasmatic membrane, anti-SAP polyclonal antibody colocalized with MAb-2C2 (Figure 3), a monoclonal antibody that reacts with carbohydrate epitopes of the parasite major surface glycoprotein Ssp-4 [28].

The differential localization of SAP proteins was further confirmed in an experiment with parasites transfected with a full-length SAP gene that encodes an N-terminal ER signal peptide and a C-terminal signal peptide for GPI anchor addition (accession number Tc00.1047053507163.30) in fusion with the green fluorescent protein (GFP). Epimastigotes were transfected with plasmid pTREX/SAP_GFP and differentiated into metacyclic trypomastigotes, which were used to infect Vero cells. Transfected extracellular amastigotes as well as epimastigotes were analyzed by immunofluorescence microscopy using an anti-GFP monoclonal antibody. The SAP_GFP protein was detected in the anterior region of epimastigotes and on the cell surface of extracellular amastigotes (Figure S1). Whether SAP proteins expressed in amastigotes are attached to the parasite surface through a GPI anchor, and what functions they play in epimastigotes and amastigotes, remains to be investigated.

### SAP proteins are released into the extracellular medium

**Figure 4:**
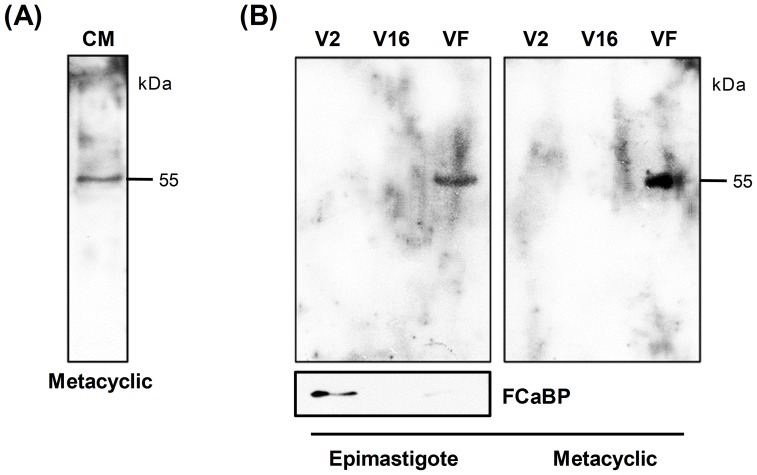
Release of SAP proteins into the extracellular medium. (A) Metacyclic trypomastigotes (CL strain) were incubated overnight in PBS at 28°C (1.0×10^8^ parasites/mL). After centrifugation, the conditioned medium (CM) was filtered and analyzed by western blot using MAb-SAP (diluted 1∶100). (B) Epimastigotes and metacyclic trypomastigotes (Dm28c) were incubated for 6 h at 28°C in DMEM or TAU3AAG (1.0×10^8^ parasites/mL), respectively. After centrifugation the conditioned medium was filtered and submitted to ultracentrifugation according to the protocol described by Bayer-Santos et al. [14]. Vesicles and soluble-protein fractions (2 μg of protein from each fraction) were analyzed by western blot using MAb-SAP (diluted 1∶100) or a monoclonal antibody against the flagellar calcium-binding protein (FCaBP). The relative molecular masses (kDa) of the immunoreactive proteins are shown on the right. V2, fraction enriched in plasma membrane-derived vesicles/ectosomes; V16, fraction enriched in exosomes; and VF, vesicle-free fraction enriched in soluble proteins.

In experiments with *T. cruzi* G and CL strains, Baida et al. [16] showed the presence of a 55 kDa SAP protein in the supernatant of metacyclic trypomastigote culture using anti-SAP antibodies. In the present study we confirmed that a 55 kDa protein is detectable by MAb-SAP in metacyclic trypomastigote conditioned medium (Figure 4A). Recently, Bayer-Santos et al. [14] performed the proteomic analysis of *T. cruzi* secretome in which two populations of extracellular vesicles (exosomes and plasma membrane-derived vesicles/ectosomes) and soluble proteins released by epimastigotes and metacyclic trypomastigotes were characterized. In eukaryotes, the classical protein secretion pathway is dependent on the presence of an N-terminal sequence (ER signal peptide) that directs the protein to the secretory pathway (endoplasmic reticulum/Golgi). Proteins that lack`` the N-terminal signal peptide can be released by `non-classical secretion pathways [29], such as vesicle-mediated secretion. Vesicles can be released by (1) fusion of multivesicular bodies with the plasma membrane and the subsequent release of exosomes, (2) shedding of plasma membrane-derived vesicles and (3) release of apoptotic bodies [30]. In this context, we decided to investigate whether SAP proteins were released as soluble proteins or associated with vesicles. Using the same protocol described previously [14], parasite conditioned medium derived from epimastigote and metacyclic trypomastigote cultures (Dm28c) was fractionated by ultracentrifugation, and total protein from plasma membrane-derived vesicles (V2), exosomes (V16) and vesicle-free fractions containing soluble proteins (VF) was analyzed by western blot using MAb-SAP. As shown in Figure 4B, a 55 kDa SAP protein was detected in vesicle-free fractions (VF) secreted by both parasite forms. In order to assess that the fractionation protocol was successful, we incubated the same epimastigote extracts with a monoclonal antibody against the flagellar calcium-binding protein (FCaBP), which was previously shown to be enriched in plasma-membrane derived vesicles (V2) [14]. Results revealed that FCaBP was mainly found in V2 fraction while SAP was enriched in VF fraction, thus confirming the differential fractionation.

SAP proteins were not reported in the analysis of *T. cruzi* secretome using mass spectrometry because only proteins identified by two distinct peptides were considered in that study [14]. Hence, it is possible that some proteins were excluded from the analysis. We therefore decided to reanalyze these proteomic data in the present study and search for SAP proteins among those identified by only one peptide. Ten peptides with similarity to SAP proteins released by epimastigotes and metacyclic forms, either as soluble proteins or associated with plasma membrane-derived vesicles (V2) and/or exosomes (V16), were identified (Table 2). In epimastigotes, three peptides were identified in V2 and two peptides in V16 and one additional peptide in the soluble fraction; whereas in metacyclic forms, two SAP peptides were identified in V2 and two peptides were in the soluble fraction (Table 2). As some peptides have high similarity with more than one SAP variant, it was not possible to establish to what SAP variant they corresponded. The reason why only SAP proteins released as soluble proteins were detected in the western blot (Figure 4B) remains to be elucidated. One possible reason is that MAb-SAP does not recognize all SAP variants, reacting predominantly with the soluble variants.

**Table 2:** SAP proteins secreted into the extracellular medium by epimastigotes and metacyclic trypomastigotes (clone Dm28c) identified by mass spectrometry.

Peptide sequence	Accession number^(1)^	Sample	Xcorr^(2)^	DC*n* ^(3)^
IEDGAEHGVFTINVSTFTQNQVK	-	Epi-V2^(4)^	2.53	0.187
INVTSPTPNILEIWWK	Tc00.1047053508219.90	Epi-V2	2.71	0.179
AVTVAIVPAKPPEVPRSPPDDK	Tc00.1047053507953.70	Epi-V2	2.79	0.114
SLWYDCTAEAGGLGSVICGMGVGNCEPEDVERR	Tc00.1047053510373.30	Epi-V16^(5)^	2.74	0.222
INVTSPTPNILEIWWK	Tc00.1047053508219.90	Epi-V16	2.72	0.194
DGAKDGVFTINVTSPNPSAVKSWWQR	Tc00.1047053506499.90	Epi-VF^(6)^	2.25	0.181
DSRDTSTPDSESPADAANSAGARSLAETPPGGAGESVPATR	Tc00.1047053510373.30	Meta-V2	2.38	0.148
GFGQMICGMGVGNCASEDFKK	Tc00.1047053504081.440	Meta-V2	2.08	0.244
KNSVSPTAGAEVAAGAPSPA	-	Meta-VF	2.02	0.197
GKPLWYNCTDAASDAGKMICDMGVGNCAPEDVEK	-	Meta-VF	2.67	0.261

(1) Accession number according to the TriTrypDB database.

(2 and 3) To validate the quality of protein identification, the following parameters were used: (2) Xcorr (CrossCorr/avg [AutoCorr offset = -75 to 75]) ≥1.5, 2.0, and 2.5, for singly, doubly and triply charged peptides, respectively. (3) DC*n* (Xcorr_1_ – Xcorr_2_/Xcorr_1_) ≥0.1.

(4) Sample enriched in plasma membrane-derived vesicles/ectosomes.

(5) Sample enriched in exosomes.

(6) Sample enriched in soluble proteins (vesicle-free, VF).

Gonçalves et al. [12] demonstrated that *T. cruzi* is continuously releasing vesicles enriched in surface antigens involved in host-cell invasion, such as glycoproteins of the GP85/TS superfamily. Since then, many proteins secreted by *T. cruzi* have been characterized [12], [13], [31]–[39]. Among these is cruzipain, the main *T. cruzi* papain-like cysteine protease, which is constitutively secreted by trypomastigotes into the extracellular medium as free cruzipain and cruzipain-chagasin complexes. Secreted cruzipain plays a role in host-cell invasion [11], [40], [41], inflammation [42], [43] and host immune system evasion [44]–[46]. Released vesicles inoculated in mice elicited an intense inflammatory response by stimulating IL-4 and IL-10 synthesis, increasing the number of amastigote nests and inducing severe heart lesion [15]. These data support the hypothesis that *T. cruzi*-secreted molecules act as important signals between the parasite and host cell and as modulators of the host immune system during infection. As several proteins identified in *T. cruzi* secretome are associated with infectivity [14], it is possible that parasite coordinates the secretion of functionally related proteins.

### SAP-CE attaches to host cell and induces lysosome exocytosis required for invasion by *T. cruzi* metacyclic forms

**Figure 5:**
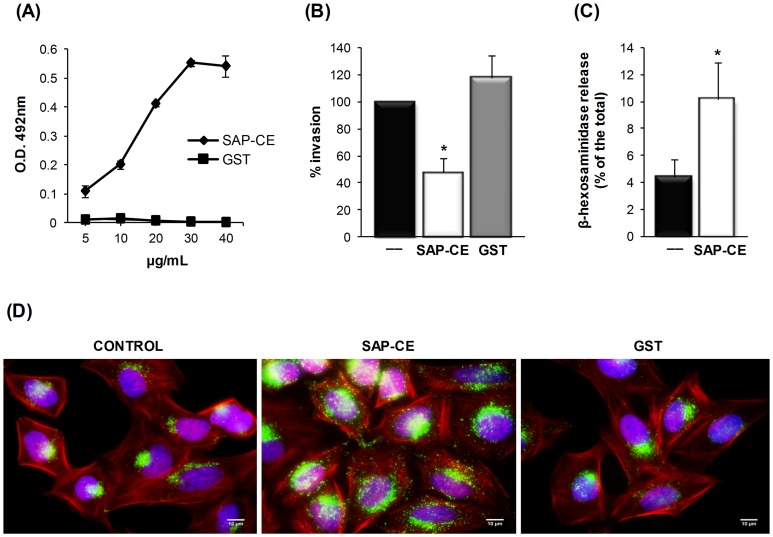
Cell adhesion and lysosome exocytosis-inducing properties of SAP-CE associated with *T. cruzi* metacyclic trypomastigote internalization. (A) Increasing amounts of the purified recombinant protein SAP-CE or GST were added to 96-well plates covered with HeLa cells. After fixation and washes in PBS, the cells were incubated with MAb-SAP (diluted 1∶100) and with anti-mouse IgG peroxidase conjugate. The bound protein was revealed by *o*-phenylenediamine. Values are the means ± standard deviations of triplicates. (B) HeLa cells were incubated for 30 min with or without the recombinant protein SAP-CE or GST (40 μg/mL) and then incubated with metacyclic forms. After incubation for 1 h, cells were washed in PBS, fixed, and stained with Giemsa. The number of internalized parasites was counted in 500 cells. The values represent the means ± standard deviations of three independent experiments performed in duplicate. SAP-CE significantly inhibited parasite invasion (*p<0.05). (C) Semi-confluent HeLa cell monolayers were incubated in absence or in the presence of GST or the purified recombinant protein SAP-CE (20 μg/mL) for 60 min. The supernatant was collected and the release of β-hexosaminidase measured. Exocytosis was expressed as a percentage of the total β-hexosaminidase activity (supernatant + cell extract). Values are the means ± standard deviations of four independent experiments performed in duplicate. β-hexosaminidase activity was significantly higher in the presence of SAP-CE (*p<0.05). (D) HeLa cells were incubated with or without the purified recombinant protein SAP-CE (20 μg/mL) and processed for indirect immunofluorescence using anti-Lamp-2 antibody and Alexa Fluor 488-conjugated anti-mouse IgG (green), phalloidin-TRITC (red) for actin visualization and DAPI (blue) for DNA. Scale bar, 10 µm.

A previous study with CL strain metacyclic forms showed that SAP proteins are implicated in host-cell invasion and that the SAP central domain (SAP-CD), which comprises 155 amino acids, is responsible for cell adhesion [16]. To further identify the region within SAP-CD involved in target cell adhesion/invasion, we generated recombinant proteins corresponding to amino-terminal (SAP-NT), central (SAP-CE) and carboxy-terminal (SAP-CT) regions of SAP-CD (Figure S2). The SAP-CE protein consisted of 54 amino acids (SAP-CD residues 61–114), shared by SAP-NT and SAP-CT proteins, of which thirty one were either serine, alanine or proline residues (Figure S2). All constructs expressed as GST fusion proteins reacted with MAb-SAP (Figure S2). To examine whether SAP-CE protein exhibited cell adhesion properties, HeLa cells were incubated with this recombinant protein at varying concentrations and bound protein was revealed with MAb-SAP. As shown in Figure 5A, SAP-CE bound to HeLa cells in a dose-dependent and saturable manner. In cell invasion assays, CL strain metacyclic forms were incubated with HeLa cells in the absence or presence of SAP-CE or GST. Parasite internalization was inhibited by 52% in the presence of SAP-CE compared with the control, which contained no recombinant protein or GST (Figure 5B).

SAP-CD shares with GP82, a metacyclic trypomastigote surface molecule, the ability to trigger a Ca^+2^ signal upon binding to HeLa cells [16]. Here, we examined whether SAP-CE, which comprises the core 54 amino acids of SAP-CD, shared with GP82 another property that is essential for promoting metacyclic trypomastigote entry into host cells, namely, the ability to induce lysosome exocytosis. HeLa cells were incubated with or without purified recombinant SAP-CE protein. After 1 h, the supernatant was collected and the activity of lysosome-specific enzyme β-hexosaminidase was measured. As shown in Figure 5C, a significant increase in lysosome exocytosis was detected in cells treated with SAP-CE. Using immunofluorescence microscopy with a monoclonal antibody to the ubiquitous lysosomal protein Lamp-2, we ascertained that lysosomes were mobilized to the cell periphery upon interaction with SAP-CE, but not with GST (Figure 5D). Lysosome distribution, similar to that induced by recombinant SAP-CE has been observed upon incubation of HeLa cells with recombinant proteins based on molecules implicated in promoting metacyclic trypomastigote internalization, such as GP30 and GP82 [47], [48]. Addition of SAP-CE protein did not result in marked pH change that could influence the lysosome mobilization. When we measured the pH of the supernatants from cells incubated for 1 h with GST or SAP-CE, the difference between them was in the 0.01–0.02 range.

To our knowledge, the metacyclic trypomastigote surface molecule GP82 and SAP proteins are the only molecularly defined *T. cruzi* components with cell adhesion capacity that have been shown to induce a Ca^+2^ signal and lysosome exocytosis, events associated with parasite internalization. A synergistic effect of SAP and GP82 in the process of host-cell invasion is an interesting possibility.

## Conclusions

Although many proteins that participate in host cell-*T. cruzi* interactions have been characterized, there are many novel proteins expressed by this parasite, whose functions are unknown and require elucidation. Taken together, our results showed that SAP proteins are released into the extracellular medium by epimastigotes and metacyclic trypomastigotes as soluble factors or as components of secreted vesicles. We also identified different sets of SAP variants that are directed to the parasite surface in extracellular amastigotes and tissue culture-derived trypomastigotes. In addition, we proposed a role for SAP proteins during internalization of metacyclic forms that relies on the interaction of the 54-amino acid SAP-CE fragment with target cells and induction of host-cell lysosome exocytosis. SAP proteins probably act synergistically with GP82 during host-cell invasion by up-regulating intracellular Ca^+2^ signaling.

## Supporting Information

Figure S1 **Expression and cellular distribution of the protein SAP_GFP in transfected parasites.** (A) Epimastigotes were transfected with a full-length SAP gene (accession number Tc00.1047053507163.30) encoding an N-terminal ER signal peptide (blue) and a C-terminal GPI anchor addition site (black) in fusion with the green fluorescent protein (GFP). Sph1 restriction site presented in the SAP sequence was used to insert the GFP gene (green). The arrows denote the annealing site of the primers used in PCR amplification and the respective restriction sites added to them. (B) Total protein extracts from epimastigotes transfected with pTREX/SAP_GFP, pTREX/GFP or untransfected controls (CL strain) were separated by electrophoresis in polyacrylamide gel, transferred to nitrocellulose membrane and incubated with anti-GFP monoclonal antibody (diluted 1∶500). The recombinant protein SAP_GFP was also recognized by MAb-SAP. (C) Epimastigotes transfected with pTREX/SAP_GFP or the control pTREX/GFP and extracellular amastigotes transfected with pTREX/SAP_GFP were fixed with 4% paraformaldehyde and incubated with anti-GFP monoclonal antibody (Sigma) diluted 1∶100, followed by incubation with Alexa Fluor 488-conjugated anti-mouse IgG (green). Parasite DNA was stained with DAPI (blue). Scale bar, 5 µm.
(TIF)

Figure S2 **Amplification by PCR of three fragments of the SAP central domain (SAP-CD).** (A) Schematic representation of the SAP-CD 513 bp fragment (accession number AF199419) and fragments SAP-NT, SAP-CE and SAP-CT amplified by PCR. The amino acid size (aa) of each fragment is shown on the right. The amino acid sequence of SAP-CE and the serine, alanine and proline residues indicated in red are also represented. (B) The purified recombinant proteins SAP-NT, SAP-CE and SAP-CT were separated by electrophoresis in a 12% polyacrylamide gel, transferred to a nitrocellulose membrane and incubated with MAb-SAP (diluted 1∶100). The antigen-antibody complexes were detected with anti-mouse IgG peroxidase conjugate (diluted 1∶5000) and revealed with DAB and hydrogen peroxide.
(TIF)

Table S1 **Full-length SAP genes identified in the** ***T. cruzi*** **genome (clone CL Brener).**
(DOCX)

Table S2 **Genomic localization of SAP sequences identified in the** ***T. cruzi*** **genome.**
(DOCX)

Table S3 **Frequency and gene content of 100 kb genomic regions containing SAP genes.**
(DOCX)
