# Supplementary figures and images for: Molecular Characterization of Trypanosoma cruzi SAP Proteins with Host-Cell Lysosome Exocytosis-Inducing Activity Required for Parasite Invasion
Source: PLoS One. 2013 Dec 31;8(12):e83864. doi: 10.1371/journal.pone.0083864 (PMC3877114; doi:10.1371/journal.pone.0083864)

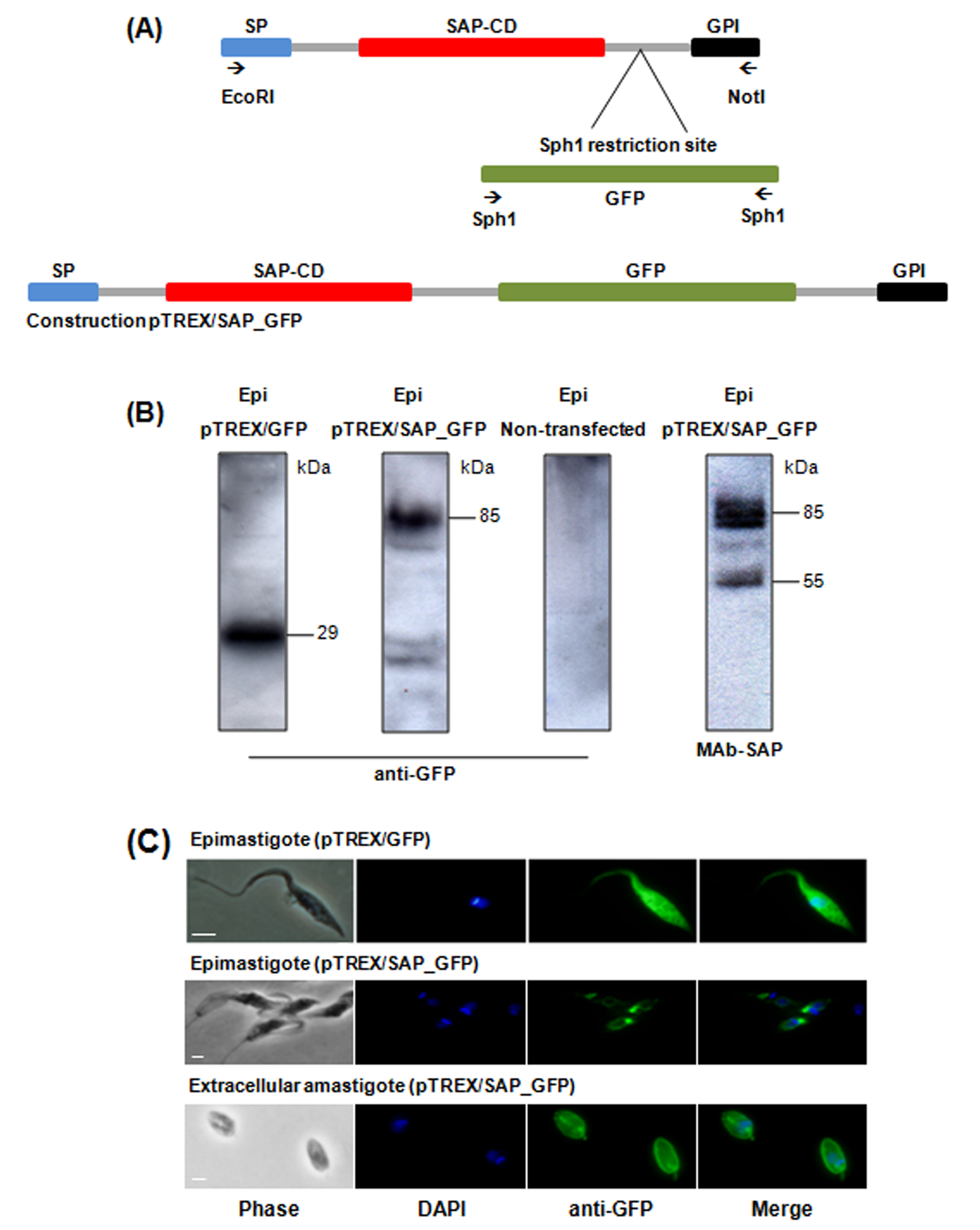

Supplement: Figure S1 — Expression and cellular distribution of the protein SAP_GFP in transfected parasites. (A) Epimastigotes were transfected with a full-length SAP gene (accession number Tc00.1047053507163.30) encoding an N-terminal ER signal peptide (blue) and a C-terminal GPI anchor addition site (black) in fusion with the green fluorescent protein (GFP). Sph1 restriction site presented in the SAP sequence was used to insert the GFP gene (green). The arrows denote the annealing site of the primers used in PCR amplification and the respective restriction sites added to them. (B) Total protein extracts from epimastigotes transfected with pTREX/SAP_GFP, pTREX/GFP or untransfected controls (CL strain) were separated by electrophoresis in polyacrylamide gel, transferred to nitrocellulose membrane and incubated with anti-GFP monoclonal antibody (diluted 1∶500). The recombinant protein SAP_GFP was also recognized by MAb-SAP. (C) Epimastigotes transfected with pTREX/SAP_GFP or the control pTREX/GFP and extracellular amastigotes transfected with pTREX/SAP_GFP were fixed with 4% paraformaldehyde and incubated with anti-GFP monoclonal antibody (Sigma) diluted 1∶100, followed by incubation with Alexa Fluor 488-conjugated anti-mouse IgG (green). Parasite DNA was stained with DAPI (blue). Scale bar, 5 µm. (TIF) [file pone.0083864.s001.tif]

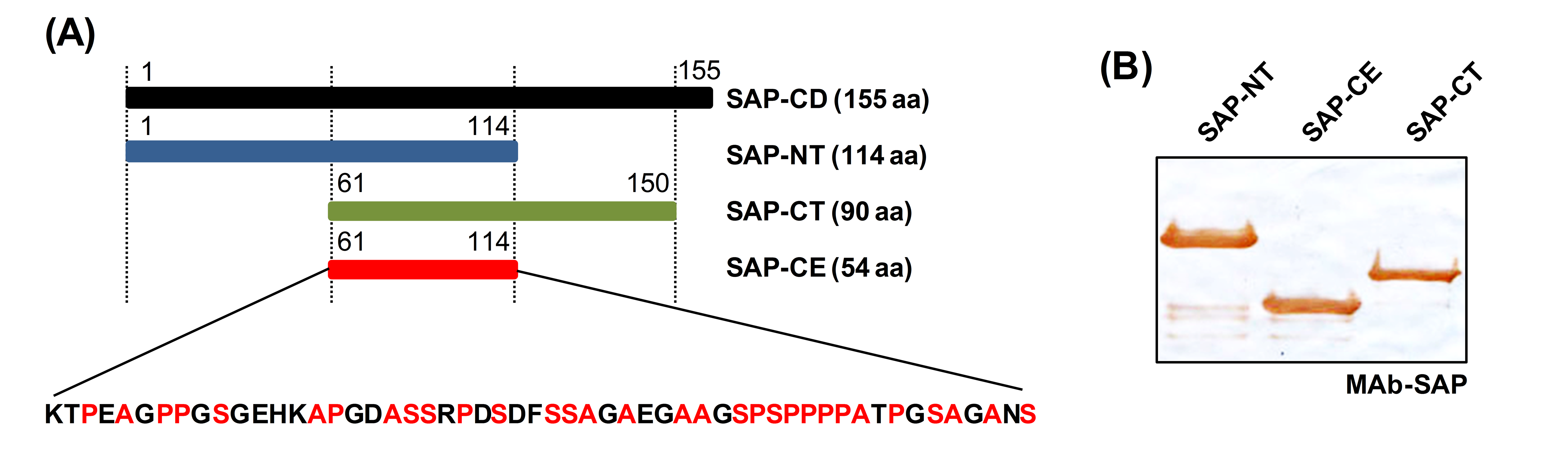

Supplement: Figure S2 — Amplification by PCR of three fragments of the SAP central domain (SAP-CD). (A) Schematic representation of the SAP-CD 513 bp fragment (accession number AF199419) and fragments SAP-NT, SAP-CE and SAP-CT amplified by PCR. The amino acid size (aa) of each fragment is shown on the right. The amino acid sequence of SAP-CE and the serine, alanine and proline residues indicated in red are also represented. (B) The purified recombinant proteins SAP-NT, SAP-CE and SAP-CT were separated by electrophoresis in a 12% polyacrylamide gel, transferred to a nitrocellulose membrane and incubated with MAb-SAP (diluted 1∶100). The antigen-antibody complexes were detected with anti-mouse IgG peroxidase conjugate (diluted 1∶5000) and revealed with DAB and hydrogen peroxide. (TIF) [file pone.0083864.s002.tif]
