# Supplementary material for: Molecular Characterization of Trypanosoma cruzi SAP Proteins with Host-Cell Lysosome Exocytosis-Inducing Activity Required for Parasite Invasion
Source: PLoS One. 2013 Dec 31;8(12):e83864. doi: 10.1371/journal.pone.0083864 (PMC3877114; doi:10.1371/journal.pone.0083864)
Supplement: Table S1 — Full-length SAP genes identified in the T. cruzi genome (clone CL Brener). (DOCX) [file pone.0083864.s003.docx]

| **ORF** | **Accession number^(1)^** | **ORF size (bp)** | **Protein size (aa)** | **TcChr^(2)^** |
| --- | --- | --- | --- | --- |
| 1 | Tc00.1047053508221.260 | 984 | 327 | TcChr41 |
| 2 | Tc00.1047053508221.730 | 1005 | 334 | TcChr41 |
| 3 | Tc00.1047053511487.150 | 993 | 330 | TcChr41 |
| 4 | Tc00.1047053508873.289 | 1017 | 338 | TcChr41 |
| 5 | Tc00.1047053504081.440 | 1020 | 339 | TcChr41 |
| 6 | Tc00.1047053508219.90 | 1182 | 393 | TcChr41 |
| 7 | Tc00.1047053505025.60 | 999 | 332 | TcChr41 |
| 8 | Tc00.1047053507981.30 | 987 | 328 | TcChr41 |
| 9 | Tc00.1047053510021.80 | 1089 | 362 | TcChr41 |
| 10 | Tc00.1047053507953.200 | 1070 | 389 | TcChr41 |
| 11 | Tc00.1047053510025.70 | 1173 | 390 | TcChr41 |
| 12 | Tc00.1047053510013.200 | 1164 | 387 | TcChr41 |
| 13 | Tc00.1047053506759.140 | 1173 | 390 | TcChr41 |
| 14 | Tc00.1047053507953.70 | 1161 | 386 | TcChr41 |
| 15 | Tc00.1047053504239.280 | 1164 | 387 | TcChr41 |
| 16 | Tc00.1047053506499.220 | 1176 | 391 | TcChr41 |
| 17 | Tc00.1047053510021.160 | 1158 | 385 | TcChr41 |
| 18 | Tc00.1047053510373.30 | 1173 | 390 | TcChr41 |
| 19 | Tc00.1047053507163.30 | 1017 | 338 | TcChr16 |
| 20 | Tc00.1047053510279.40 | 1020 | 339 | TcChr40 |
| 21 | Tc00.1047053510483.280 | 1029 | 342 | TcChr38 |
| 22 | Tc00.1047053510693.40 | 1185 | 394 | TcChr38 |
| 23 | Tc00.1047053506667.30 | 1008 | 335 | TcChr20 |
| 24 | Tc00.1047053508099.20 | 990 | 329 | TcChr20 |
| 25 | Tc00.1047053511233.160 | 1005 | 334 | TcChr18 |
| 26 | Tc00.1047053508247.110 | 1005 | 334 | TcChr18 |
| 27 | Tc00.1047053508853.30 | 1005 | 334 | TcChr18 |
| 28 | Tc00.1047053511599.30 | 942 | 313 | TcChr18 |
| 29 | Tc00.1047053511603.470 | 933 | 310 | TcChr18 |
| 30 | Tc00.1047053511605.55 | 939 | 312 | TcChr18 |
| 31 | Non-annotated | 1095 | 392 | Tcruzi_7398 |
| 32 | Non-annotated | 1179 | 364 | Tcruzi_21632 |
| 33 | Tc00.1047053504081.250 | 1062 | 353 | TcChr41 |
| 34 | Tc00.1047053508633.130 | 888 | 295 | Tcruzi_7809 |
| 35 | Non-annotated | 813 | 270 | Tcruzi_3010 |
| 36 | Non-annotated | 822 | 273 | Tcruzi_30816 |
| 37 | Tc00.1047053511553.160 | 855 | 284 | TcChr41 |
| 38 | Tc00.1047053506499.90 | 1326 | 441 | TcChr41 |
| 39 | Tc00.1047053506499.190 | 1740 | 579 | TcChr41 |

(1) Accession number according to the TriTrypDB database.

(2) Localization of SAP genes based on the 41 chromosome-sized scaffolds [25].
