# Supplementary material for: Molecular Characterization of Trypanosoma cruzi SAP Proteins with Host-Cell Lysosome Exocytosis-Inducing Activity Required for Parasite Invasion
Source: PLoS One. 2013 Dec 31;8(12):e83864. doi: 10.1371/journal.pone.0083864 (PMC3877114; doi:10.1371/journal.pone.0083864)
Supplement: Table S2 — Genomic localization of SAP sequences identified in the T. cruzi genome. (DOCX) [file pone.0083864.s004.docx]

| **Chromosomal platforms**  **(TcChr)^(1)^** | **Number of SAP genes** |
| --- | --- |
| TcChr41 | 26 |
| TcChr18 | 6 |
| TcChr40 | 3 |
| TcChr38 | 2 |
| TcChr20 | 2 |
| TcChr16 | 1 |
| Non-allocated contigs | 11 |
| **TOTAL** | **51** |

(1) Chromosome-sized scaffolds defined by Weartherly et al. [25].
