# Supplementary material for: Molecular Characterization of Trypanosoma cruzi SAP Proteins with Host-Cell Lysosome Exocytosis-Inducing Activity Required for Parasite Invasion
Source: PLoS One. 2013 Dec 31;8(12):e83864. doi: 10.1371/journal.pone.0083864 (PMC3877114; doi:10.1371/journal.pone.0083864)
Supplement: Table S3 — Frequency and gene content of 100 kb genomic regions containing SAP genes. (DOCX) [file pone.0083864.s005.docx]

| **Gene^(1)^** | **Number of genes** | | **%** |
| --- | --- | --- | --- |
| Mucin-associated surface protein (MASP) | 325 | | 28.5 |
| Hypothetical protein | 313 | | 27.4 |
| Mucin | 175 | | 15.3 |
| Trans-sialidase | 140 | | 13.0 |
| Surface protease GP63 | 74 | | 6.5 |
| Syntaxin binding protein | 27 | | 2.4 |
| Elongation factor 1-gamma (EF-1-gamma) | 25 | | 2.2 |
| Dispersed gene family protein 1 (DGF-1) | 20 | | 1.8 |
| Retrotransposon hot spot protein (RHS) | 20 | | 1.8 |
| Surface antigen TASV-B1 | 3 | | 0.26 |
| NADH-dependent fumarate reductase | 2 | | 0.18 |
| 40S ribosomal protein S27, putative | 2 | | 0.08 |
| Beta galactofuranosyl glycosyltransferase | 1 | | 0.08 |
| Cysteine synthase | 1 | | 0.08 |
| 2-oxoglutarate dehydrogenase E1 component | 1 | | 0.08 |
| Calmodulin, putative | 1 | | 0.08 |
| NUDIX hydrolase, putative | 1 | | 0.08 |
| Cleavage and polyadenylation specificity factor 30kDa subunit, putative | 1 | | 0.08 |
| **TOTAL** | | **1140** | **100** |

(1) Gene annotation according to the TritrypDB database.
